# Genetic disruption of calpain-1 and calpain-2 attenuates tumorigenesis in mouse models of HER2+ breast cancer and sensitizes cancer cells to doxorubicin and lapatinib

**DOI:** 10.18632/oncotarget.26078

**Published:** 2018-09-07

**Authors:** James A. MacLeod, Yan Gao, Christine Hall, William J. Muller, Taranjit S. Gujral, Peter A. Greer

**Affiliations:** ^1^ Department of Pathology and Molecular Medicine, Queen's University, Kingston, Ontario, Canada; ^2^ Division of Cancer Biology and Genetics, Queen's Cancer Research Institute, Kingston, Ontario, Canada; ^3^ Rosalind and Morris Goodman Cancer Centre, Department of Biochemistry, McGill University, Montreal, Quebec, Canada; ^4^ Division of Human Biology, Fred Hutchinson Cancer Research Center, Seattle, WA, USA

**Keywords:** calpain, capns1, breast cancer, HER2

## Abstract

Calpains are a family of calcium activated cysteine proteases which participate in a wide range of cellular functions including migration, invasion, autophagy, programmed cell death, and gene expression. Calpain-1 and calpain-2 isoforms are ubiquitously expressed heterodimers composed of isoform specific catalytic subunits coupled with an obligate common regulatory subunit encoded by *capns1*. Here, we report that conditional deletion of capns1 disrupted calpain-1 and calpain-2 expression and activity, and this was associated with delayed tumorigenesis and altered signaling in a transgenic mouse model of spontaneous HER2^+^ breast cancer and effectively blocked tumorigenesis in an orthotopic engraftment model. Furthermore, *capns1* knockout in a tumor derived cell line correlated with enhanced sensitivity to the chemotherapeutic doxorubicin and the HER2/EGFR tyrosine kinase inhibitor lapatinib. Collectively, these results indicate pro-tumorigenic roles for calpains-1/2 in HER2^+^ breast cancer and provide evidence that calpain-1/2 inhibitors could have anti-tumor effects if used either alone or in combination with chemotherapeutics and targeted agents.

## INTRODUCTION

Calpains are a family of 15 calcium-dependent intracellular thiol proteases which are involved in a wide range of cellular and physiological functions (reviewed in [1–3]). Aberrant expression or activity of calpains is implicated in the etiology of several diseases including diabetes, Alzheimer's and cancer; this has stimulated considerable interest in the development and preclinical testing of calpain inhibitors (reviewed in [4]); however, to date there are none approved for clinical use. Calpain-1 and calpain-2, the most widely studied members of this family, are ubiquitously expressed heterodimers composed of isoform specific catalytic subunits encoded by *capn1* or *capn2*, respectively, coupled with an obligate common regulatory subunit encoded by *capns1*. Genetic disruption of *capns1* is associated with destabilization of the CAPN1 and CAPN2 catalytic subunits and loss of calpain-1 and calpain-2 activities *in vivo* [5, 6].

Hundreds of calpain substrates have been described [7], and the effect of calpain cleavage on their functions (where studied) varies from activation to inactivation and often involves changes in subcellular localization or protein-protein associations. Since many of these substrates are components of key cell signaling pathways, calpains can have pleomorphic effects on cellular behavior, depending upon cell type, substrate expression and the context in which calpain is activated. Many of the pathways calpain activity impinges upon are related to tumorigenesis (reviewed in [8]), including key survival and apoptosis pathways such as the PI3K-AKT pathway [9–11], cell cycle checkpoints [12–14], migration and invasion [15–18], as well as the function of oncoproteins such as HER2 [19] and MYC [20]. As calpain function is implicated in diverse signaling networks, perhaps unsurprisingly, there is evidence that calpain may engage in opposing roles; for example, promoting apoptosis in response to challenge with etoposide or camptothecin, and protecting the cell from cytotoxic responses to tumor necrosis factor α or staurosporine [11].

Translational studies have revealed that high calpain-2 expression correlates with adverse outcomes in basal-like or triple-negative breast cancer [21], while high calpain-1 expression correlated with poor relapse-free survival in HER2^+^ breast cancer [22]. High calpain-2 levels were also associated with platinum resistance and poor overall survival in ovarian cancer patients [23].

*In vitro* studies have linked calpain to trastuzumab resistance in HER2^+^ breast cancer cells through generation of a p95HER2 fragment [19, 24], or resistance to chemotherapeutics like doxorubicin through regulating multidrug resistance protein function [25]. Thus, a growing body of research suggests that inhibition of calpain may suppress tumorigenesis and could cooperate or synergize with specific existing treatments to improve breast cancer patient outcomes.

In this study we use genetic manipulation of *capns1* in HER2^+^ models of breast cancer to show that calpain-1 and/or calpain-2 are involved in but not required for spontaneous tumor formation in a transgenic mouse model of HER2/NEU-driven tumorigenesis; however, *capns1* knockout in established carcinoma cells effectively blocked their tumor forming capability in an orthotopic engraftment model and enhanced *in vitro* sensitivity to doxorubicin and lapatinib.

## RESULTS

### Deletion of *capns1* delays HER2/NEU-induced tumorigenesis

**Figure 1:**
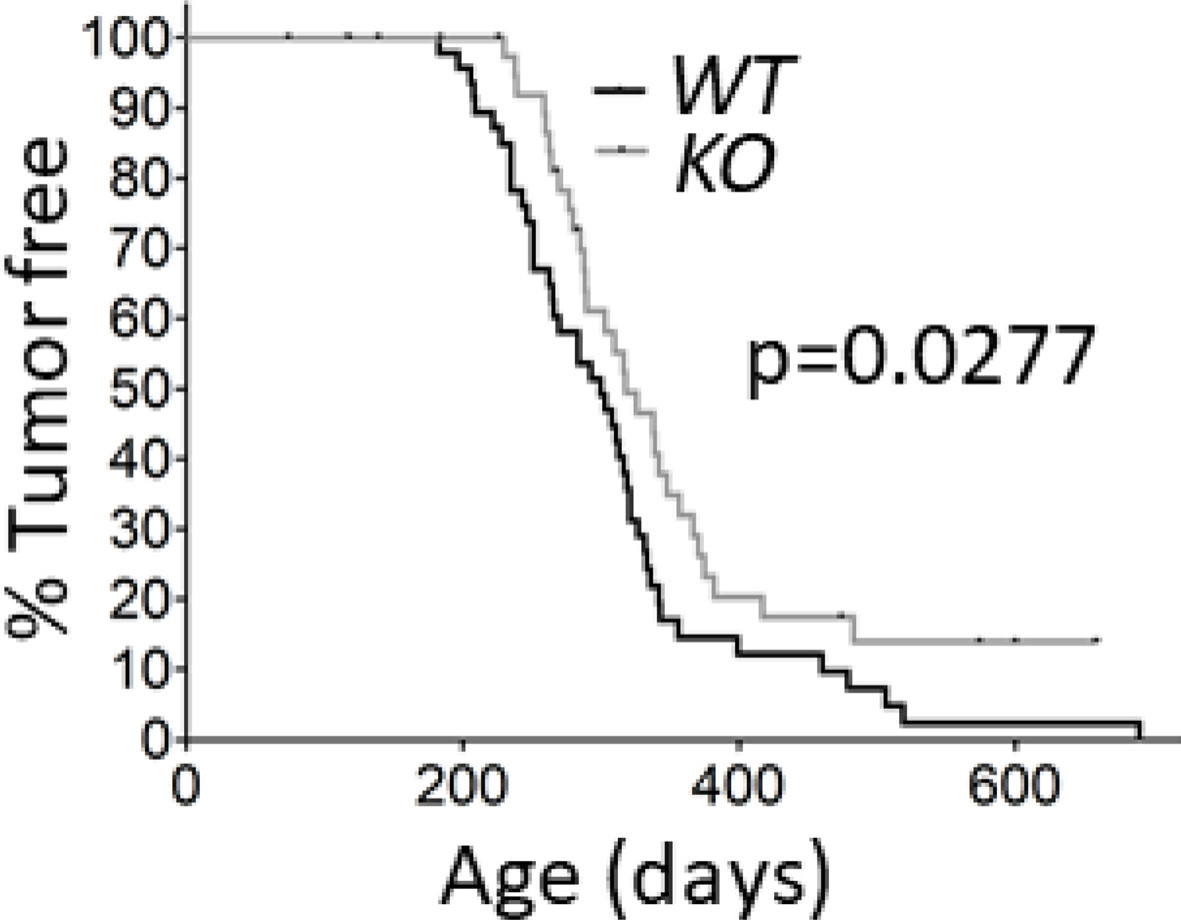
Deletion of *capns1* in the mammary epithelium delays Her2/*Neu*-driven tumorigenesis *NIC capns1*^flox/flox^ (*KO*) or *NIC capns1^+/+^* (*WT*) female mice were assessed for tumor onset by weekly palpitation. Median tumor onset was 318 vs 300 days, (*n* = 43 vs 48, respectively, *p* = 0.0277^*^ Gehan-Breslow-Wilcoxon Test).

The stability and activity of calpain-1 and calpain-2 are contingent upon expression of the common regulatory subunit encoded by *capns1* [26, 27], and *capns1* knockout in transgenic mice has been shown to abolish both calpain-1 and calpain-2 [5, 6]. To test the potential involvement of calpain-1 and calpain-2 in HER2-driven mammary tumorigenesis, we crossed the *NIC* transgenic mouse model [28] with conditionally targeted (*floxed*) *capns1* mice [6]. Co-expression of oncogenic HER2/NEU and the CRE recombinase from the *NIC* transgene in the mammary epithelium resulted in deletion of *floxed capns1* alleles and ablation of CAPNS1 expression in mammary tumors arising in *NIC capns1^flox/flox^* (KO) mice, while tumors from *NIC capns1^+/+^* (WT) mice retained CAPNS1 expression (Supplementary Figure 1). Deletion of *capns1* in the mammary epithelium correlated with a significant delay in spontaneous tumor onset (median time KO = 318 vs WT = 300 days; *p* = 0.0277); and 10% of KO mice remained tumor-free beyond 600 days of age while nearly all WT mice had developed tumors by this age (Figure 1). While these data show that calpain-1 and calpain-2 are not necessary for HER2/NEU-driven tumorigenesis, they indicate that one or both calpains contribute to carcinogenesis.

**Figure 2:**
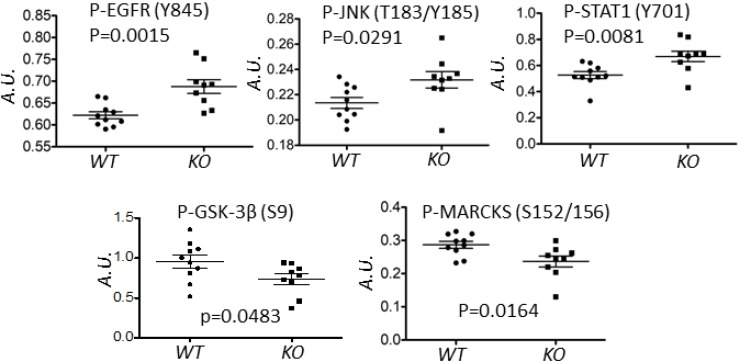
Deletion of *capns1* in Her2/*Neu*-driven mouse mammary tumors correlates with a differential phosphoproteome Spontaneous tumors arising in *NIC capns1*^flox/flox^ (*KO*, *n* = 9) or *NIC capns1^+/+^* (*WT*, *n* = 10) female mice were subject to RPPA analysis with 128 antibodies. The indicated phosphoproteins displayed significantly different signal intensities (A.U.).

To assess possible calpain-mediated signaling pathways that underpin this delay, an RPPA analysis was performed on tumor lysates using 128 antibodies, predominantly directed against phosphopeptides in important signaling nodes [29, 30]. This analysis revealed five proteins (EGFR, JNK, STAT1, MARCKS and GSK3β) which were differentially phosphorylated in WT and KO tumors (Figure 2).

### Establishment of a conditional *capns1* knockout HER2/NEU-driven mammary tumor epithelial cell line

**Figure 3:**
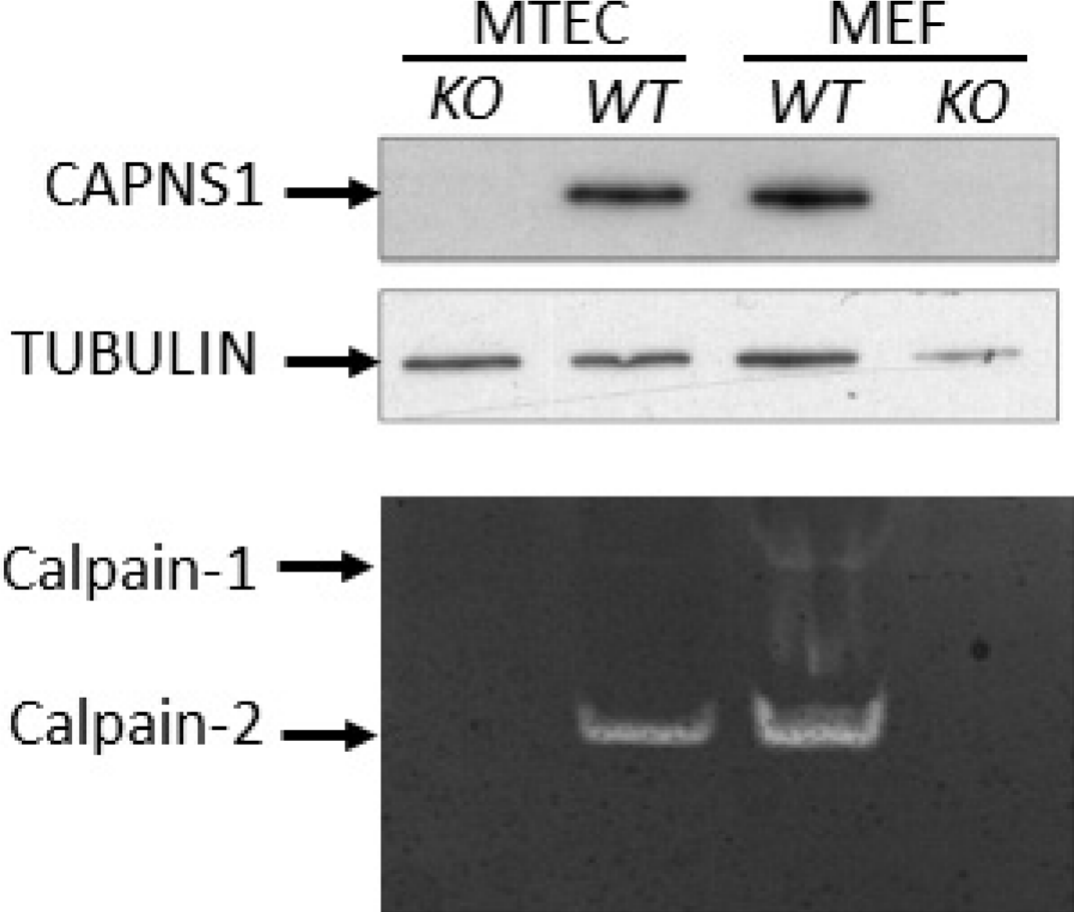
CAPNS1 protein and calpain-1/2 activities are ablated by *capns1* deletion (**Upper**) Lysates from *capns1^flox/flox^* MTECs transduced with Cre-expressing (*KO*) or control (*WT*) retroviruses; or *capns1^+/+^* (*WT*) or *capns1*^−*/*−^ (*KO*) mouse embryonic fibroblasts (MEFs) were subjected to immunoblotting analysis with antibodies recognizing CAPNS1 or tubulin. (**Lower**) Casein zymography assessment of calpain-1 and calpain-2 activities in lysates from the same panels of MTEC and MEF cells.

To further study the involvement of calpains-1/2 in HER2/NEU-driven tumorigenesis we next established a conditional *capns1* knockout mammary carcinoma cell line model. *Floxed capns1* mice were crossed with *neu^NT^* transgenic mice [31], which express oncogenic HER2/NEU in the mammary epithelium under the control of the MMTV LTR. A tumor arising in a *neu^NT^ capns1^flox/flox^* female mouse was used to derive a mammary tumor epithelial cell (MTEC) line. To create isogenic MTECs without or with calpain-1/2 activity, *capns1^flox/flox^* MTECs were transduced with retroviruses encoding either CRE-recombinase (KO) or the empty vector (WT), respectively. The retrovirus also encoded puromycin phosphotransferase, which enabled selection of transduced cell populations. Immunoblotting of the puromycin selected populations revealed the expected loss of CAPNS1 protein expression in CRE-transduced cells, and casein zymography showed the loss of both calpain-1/2 activities (Figure 3). Mouse embryonic fibroblasts (MEFs) generated from *capns1^+/+^* (WT) and *capns1^-/-^* (KO) embryos served as controls in this analysis [6]. MTECs express predominantly calpain-2, with only trace amounts of calpain-1.

### *Capns1* deletion in MTECs did not affect *in vitro* migration but did attenuate *in vitro* invasion

Calpains-1/2 have been implicated in membrane-cytoskeletal dynamics associated with cell migration and invasion [18, 32]. *In vitro* migration and invasion were both compromised by *capns1* KO in mouse embryonic fibroblasts [15, 17] or *capns1* knockdown in MDA-MB-231 breast cancer cells [25]; and *capn2* knockdown was associated with dysregulated focal adhesion turnover [18] and lamellipodia dynamics [16] in fibroblasts, as well as attenuated migration and invasion in mammary carcinoma cells [9].

**Figure 4:**
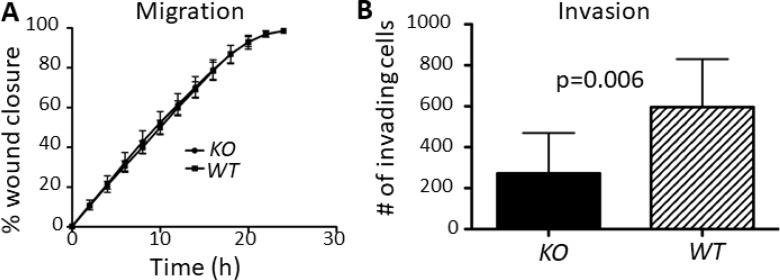
*capns1* KO is associated with attenuated MTEC *in vitro* invasion but no effect on migration (**A**) Temporal profiles of *capns1 KO* and *WT* MTEC migration behavior in scratch-wound healing assays. Fifteen thousand MTECs were seeded on ImageLock 96-well plates and confluent monolayers were scratch-wounded after an overnight incubation. Migration was monitored in an IncuCyte system for 24 h. Data are the means ± SD of three independent experiments run in sextuplicate. (**B**) Boyden chamber assays measured *capns1 KO* and *WT* MTEC invasion after 24 h through 8 μm pores of transwells coated with 15 μg of matrigel. Data are means ± SD of two independent experiments run in triplicate (*p* = 0.006, student's *t*-test).

Surprisingly, *capns1* KO MTECs did not display a migration defect in a wound healing assay (Figure 4A). However, when the invasive ability of MTECs was assessed using matrigel-coated transwell Boyden chamber assays, *capns1* KO correlated with attenuated cell invasion activity (Figure 4B, *p* = 0.006).

### *Capns1* KO sensitizes MTECs to doxorubicin and lapatinib challenge

**Figure 5:**
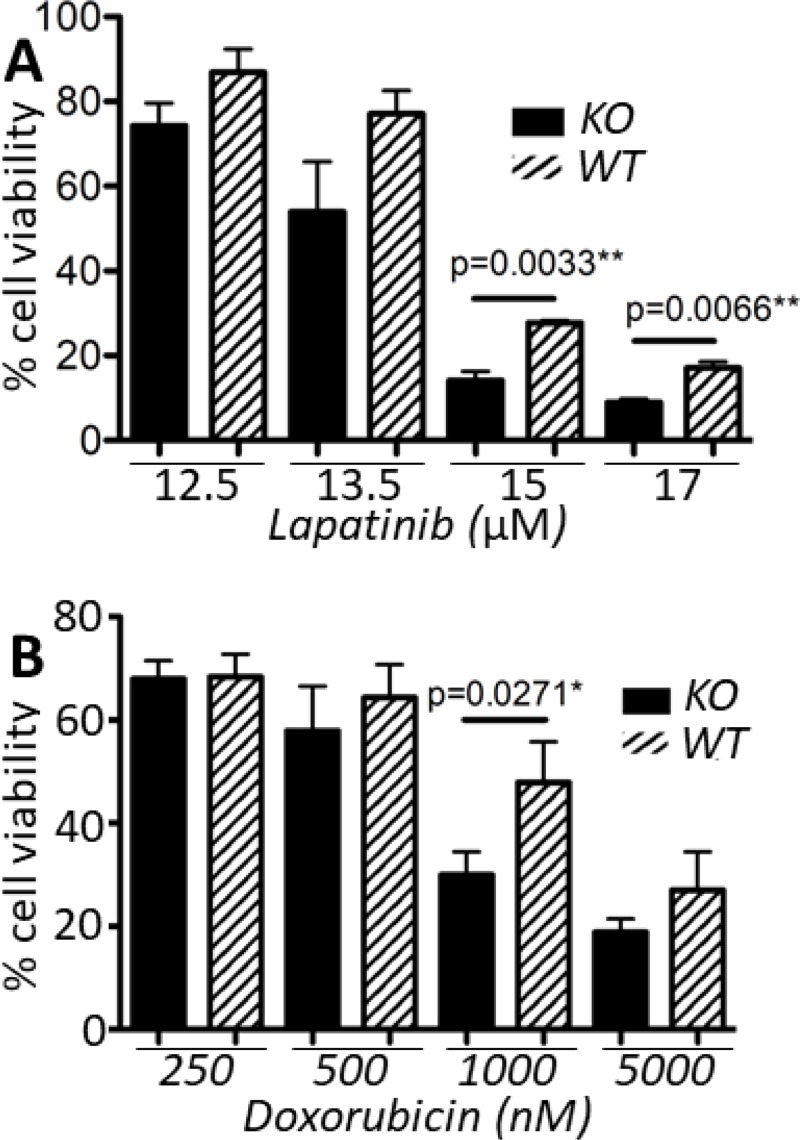
*capns1* KO correlates with enhanced sensitivity to lapatinib and doxorubicin Two × 10^4^ *capns1 KO* or *WT* MTECs were seeded on 96 well plates, then cultured overnight. The following day, cells were treated for 72 h with (**A**) lapatinib or (**B**) doxorubicin at the indicated concentrations. Cell viability was assessed at endpoint using the PrestoBlue viability reagent. Data are means ± SD of three independent experiments with experimental triplicates (*p*, student's *t*-test).

Lapatinib is a small molecule tyrosine kinase inhibitor of both HER2 and EGFR. It has similar clinical efficacy to trastuzumab in a neoadjuvant setting [33], and may also be used as a second-line therapy when resistance to trastuzumab develops [34, 35]. Doxorubicin is an anthracycline which causes DNA damage by targeting topoisomerase II and is widely used as a cancer therapeutic. Doxorubicin resistance is mediated in part by members of the ABC family of transporters including P-glycoprotein (Pgp) and multidrug resistance protein 2 (MRP2). We recently reported that expression of Pgp and MRP2, and sensitivity to doxorubicin correlate with calpain-1/2 expression [25]. Furthermore, cleavage of topoisomerase IIα by calpain-2 has been suggested as an additional mechanism of doxorubicin resistance [36]. Thus, we sought to determine whether loss of calpain-1/2 in a HER2^+^ tumor cell line model might cooperate with therapeutic challenge from these two commonly used treatments of breast cancer. Lapatinib exposure for 72 h across a range of doses was associated with a trend towards reduced viability in *capns1* KO MTECs, with significantly reduced viability at both 15 μM and 17 μM (*p* = 0.0033 and *p* = 0.0066, respectively) (Figure 5A). Upon challenging *capns1* KO or WT MTECs with doxorubicin for 72 h, a similar trend towards increased cytotoxicity in KO MTECs was observed, with significantly reduced viability noted at 1000 nM (*p* = 0.0271) (Figure 5B).

### Deletion of *capns1* blocks MTEC tumorigenesis in an orthotopic engraftment model

**Figure 6:**
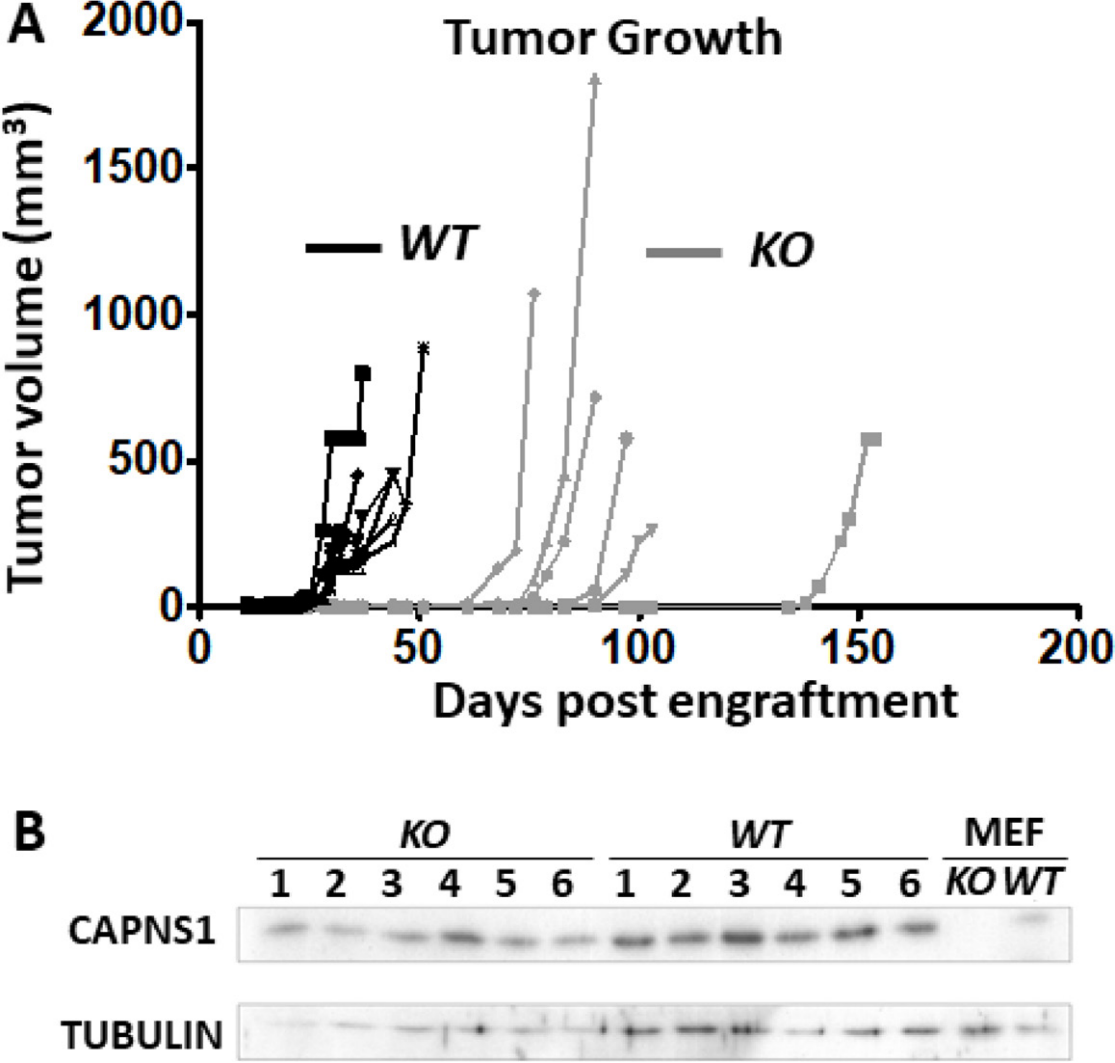
*capns1 KO* is associated with reduced MTEC tumorigenic potential One × 10^6^ *capns1 KO* or *WT* MTECs were engrafted into the number four mammary glands of female *Rag2*^−*/*−^ *IL-2Rgc*^−*/*−^ mice (*n* = 6 for each cohort). (**A**) Caliper measurements of tumor volumes. Median time to onset of palpable tumors at the engraftment site were 79 or 29 days for *KO* and *WT*, respectively (*p* = 0.0009^***^, Log-rank (Mantel-Cox) test). (**B**) Late arising tumors in mice engrafted with *capns1 KO* MTECs display CAPNS1 expression. Immunoblot analysis of tumor lysates reveals CAPNS1 expression in tumors from both *capns1 WT* and *KO* engrafted MTECs. Lysates from *WT* and *KO* MEF cells served as positive and negative controls for CAPNS1. Immunoblotting for tubulin confirmed comparable sample loading.

Previously, we showed that *shRNA*-mediated knockdown of *capn2* in murine mammary carcinoma engraftment model [9] or knockdown of *capns1* in a human triple negative breast cancer xenograft model [25] attenuated tumor growth at the orthotopic site. Here, using the *floxed capns1* HER2/NEU-driven MTEC carcinoma model, we asked if complete ablation of calpain-1 and calpain-2 would result in a more effective block in tumorigenesis in an orthotopic engraftment model. WT MTECs produced rapidly growing tumors with a mean onset time of 29 days, while tumor onset was delayed to a mean of 79 days after engraftment with KO MTEC cells, at which point KO tumors grew at comparable rates to WT tumors (Figure 6A). When resected and assessed by immunoblotting, comparable levels of CAPNS1 were observed in both WT and KO tumors (Figure 6B). Since engrafted KO cells showed no detectable CAPNS1 in immunoblotting (Figure 3), this indicates that KO tumors arose from rare “escaper” WT MTECs which had not undergone CRE-mediated deletion of the *floxed capns1* alleles and required several more weeks to emerge as palpable tumors. Delayed tumor growth behavior was validated in an independent experiment with essentially identical results (Supplementary Figure 2).

Tumor progression can be limited by failure to drive *de novo* angiogenesis in response to hypoxia due to an inability to trigger an angiogenic switch [37]. Given that engrafted *capsn1 KO* MTECs failed to develop palpable tumors, we next explored the possibility that calpain-1/2 deficient cells might not be able to respond *in vivo* to hypoxia during tumor growth. Hypoxia-inducible factor 1 alpha (HIF1α) is a transcription factor that is normally degraded by the proteasome via hydroxylation by prolyl hydroxylases under normoxic conditions, but is stabilized under hypoxic conditions to direct transcription of genes containing pro-survival hypoxic response elements including VEGF, which is needed to promote tumor-associated neoangiogenesis [38]. Calpain has been shown to modulate a hypoxic response through generation of a filamin-A fragment which mediates nuclear localization of HIF1α [39]. To test the involvement of calpain in HIF1α regulation in MTECs, we compared HIF1α levels in whole cell lysates and nuclear extracts from *capns1* KO and WT cells cultured at either hypoxic (0.1%) or ambient (20%) oxygen concentrations for 24 hours in the absence of serum. Cobalt chloride was used as a positive control for HIF1α stabilization. Hypoxia or CoCl_2_ stabilized comparable levels of HIF1α in WT and KO MTECs in both whole cell lysates and nuclear fractions (Supplementary Figure 3), suggesting that calpain does not play a key role in the angiogenic switch in this model system.

The production and activation of matrix metalloproteases (MMPs) has also been linked to tumor growth, invasion and metastasis [40–42]. Gelatin zymography analysis of conditioned media from *capns1* KO and WT MTECs revealed abundant levels of MMP2 and lower levels of MMP9, but there were no differences between the WT and KO MTECs (Supplementary Figure 4).

### *capns1* KO in MTECs correlates with enhanced EGFR expression and sustained EGF response

**Figure 7:**
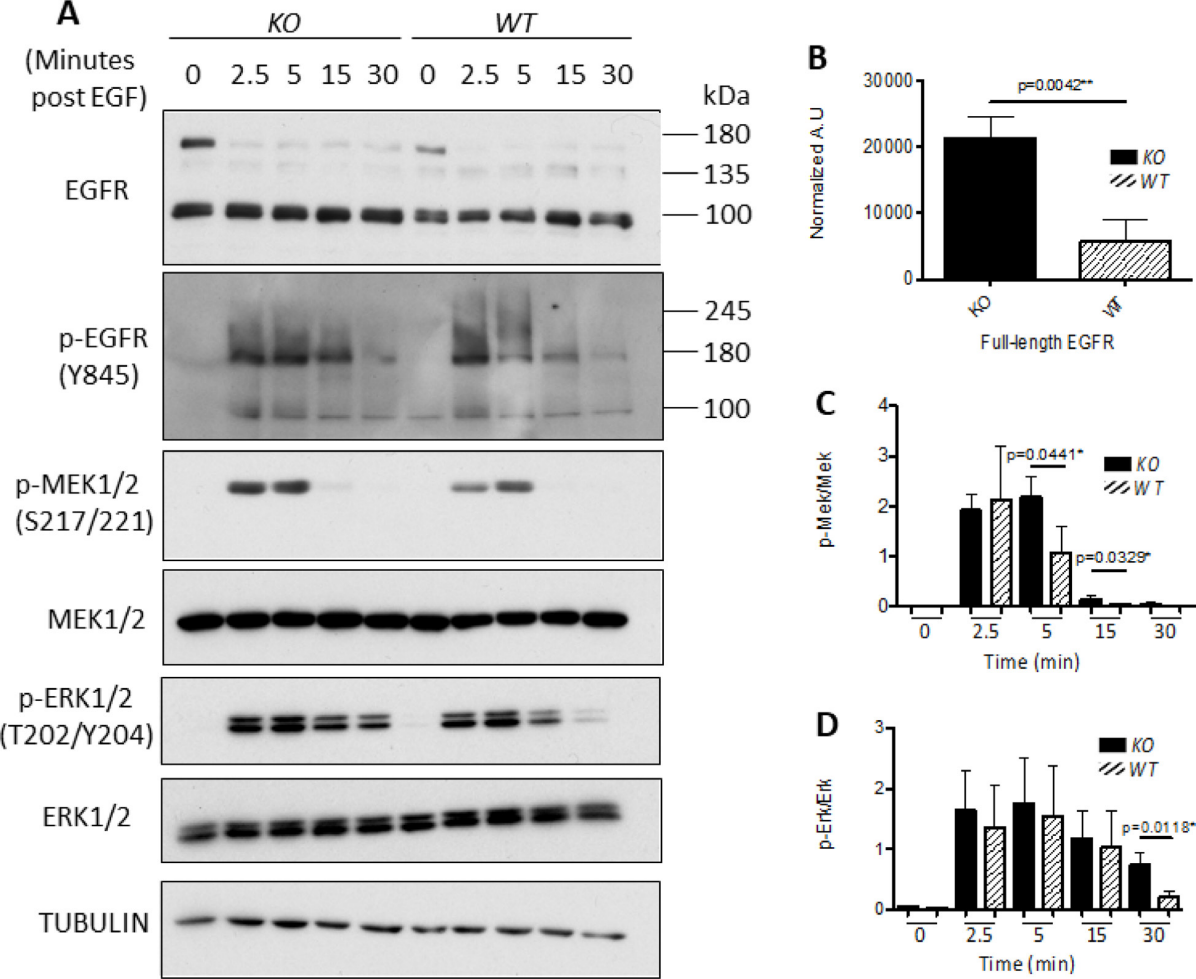
Knockout of calpain correlates with enhanced EGFR expression and sustained MAPK signaling 7.5 × 10^4^ WT or *capns1* KO MTECs were seeded overnight in 60 mm dishes. The following day cells were washed with PBS and changed to serum-free media. MTECs were stimulated with 50 ng/mL of EGF for the indicated times, lysed in RIPA buffer and assessed by immunoblotting. (**A**) c*apns1* KO correlates with activation of the MAPK pathway in response to EGF. (**B**) Full length EGFR levels are elevated in *capns1* KO MTECs after overnight serum starvation. (**C**) cap*ns1* KO is associated with greater EGF-induced MEK1/2 phosphorylation. (**D**) *capns1* KO is associated with greater EGF-induced ERK1/2 phosphorylation.

EGFR is a key dimerization partner of HER2, and is frequently expressed in breast carcinomas [43]. Deletion of *capns1* was associated with enhanced phosphorylation of EGFR (Y845) in tumors from the *NIC* model (Figure 2); we therefore tested EGF-induced activation of EGFR *in vitro* in the MTEC system. Overnight serum starvation followed by EGF stimulation resulted in a rapid induction in pY845-EGFR levels in both *capns1* KO and WT MTECs (Figure 7A), however there was a difference in kinetic behavior, with more sustained EGFR activation in the *capns1* KO MTECs; which correlated with more robust and sustained activation of MEK and ERK, as shown by their phosphorylation at activation loop sites (Figure 7A, 7C, 7D). We also noted an increase in steady-state levels of EGFR in the *capns1* KO MTECs (Figure 7B). These data are consistent with a role for calpains-1/2 in EGFR signaling to the RAS-MAPK pathway.

## DISCUSSION

Approximately 15–20% of newly diagnosed breast cancers are the highly aggressive HER2^+^ subtype [44], and despite treatment with chemotherapy and targeted therapeutics such as trastuzumab and lapatinib, half of these patients relapse with resistant disease within one year [45]. These clinical findings underscore the urgent need to develop more effective treatments for HER2^+^ breast cancer. This will require a better understanding of the biology of these tumors, including the signaling pathways that confer resistance to current treatments and identification of druggable components of these pathways that might represent novel combinatorial therapeutic targets.

Our previous work has shown that the ubiquitously expressed calpain-1 and -2 isoforms regulate cell survival in response to cytotoxic challenges [10, 11] and promote breast cancer cell line resistance to chemotherapeutics including doxorubicin and cisplatin [25]. We also showed that *capn2* knockdown in a mouse mammary carcinoma cell line was associated with reduced tumor growth in a mouse orthotopic engraftment model [9], and *capns1* knockdown in the MDA-MB-231 triple negative breast cancer cell line correlated with reduced tumor growth and metastasis in a mouse orthotopic xenograft model, and this effect was enhanced by combination with the HSP90 inhibitor, 17-AAG [25].

The relationship between expression of calpains and clinical outcomes in various cancers are being explored [8, 46]. Of specific relevance to this study, CAPN1 expression in HER2^+^ breast cancer has been positively correlated with shorter relapse-free survival and resistance to trastuzumab [22]. This is consistent with *in vitro* studies showing that *capns1* expression was associated with reduced trastuzumab sensitivity in the HER2^+^ human breast cancer cell lines SKBR3 and BT474 [47].

Here we report the first study exploring the involvement of ubiquitously expressed classical calpain-1/2 isoforms in a genetically engineered mouse model of spontaneous tumorigenesis driven by the HER2/NEU oncogene. By deleting *capns1* in the mammary epithelium, we show that calpains-1/2 are not required for HER2/NEU tumorigenesis, but their absence is associated with a significant delay in tumor onset (Figure 1). Phosphoproteomic analysis of these tumors was performed to explore the possibility that differences in the carcinogenic process in the presence or absence of calpains-1/2 might be revealed by distinct activation states of specific signaling nodes. This analysis detected significant differences in 5 phosphoproteins (EGFR, GSK-3β, JNK, MARCKS and STAT-1) out of the 128 proteins queried (Figure 2), suggesting that the carcinogenic program pursued by these HER2/NEU-driven tumors was subtly different in the absence of calpains-1/2.

Glycogen synthesis kinase 3 β (GSK-3β) has complex roles in cell signaling, including a tumor suppressor function through inhibition of the β-catenin complex [48]. GSK-3β is inhibited by AKT-mediated S9 phosphorylation and activated by PP2A-mediated dephosphorylation. Reduced p-S9-GSK-3β levels in *capns1* KO tumors is consistent with previous observations of reduced AKT activation in *capns1* KO MEF cells [11], and increased PP2A levels in *capn2* knockdown mammary carcinoma cells [9] and *capns1* KO MEFs [49]. Thus, a GSK-3β tumor suppressor role could contribute to delayed tumor onset in the *NIC capns1* KO mice.

The c-Jun N-terminal kinase (JNK) is activated in response to a number of ligands and stress stimuli and subsequently regulates transcription, migration and apoptosis [50]. Increased pT193/Y185-JNK in *capns1* KO tumors was surprising since *capns1* KO MEF cells had been shown to display reduced activation in response to endoplasmic reticulum stress [10]. This difference may reflect distinct effects of calpain deficiency in cancer cells and immortalized fibroblasts, or distinct context-specific roles of calpain in endoplasmic reticulum stress and tumorigenesis.

Calpain-mediated cleavage of protein kinase C (PKC) is an evolutionally conserved mechanism for PKC activation [51], and PKCβ deletion was correlated with suppressed tumorigenesis in a transgenic mouse model of breast cancer [52]. Myristoylated alanine-rich protein kinase C substrate (MARCKS) displayed reduced pSer-152/S162 levels in *capns1* KO tumors, consistent with a role for calpain in PKC activation in these tumors. Elevated p-Ser-152/156-MARCKS levels have been positively associated with more aggressive breast cancer and reduced sensitivity to chemotherapy in breast cancer cell lines [53]. These observations suggest that reduced calpain-mediated activation of PKC may have also contributed to delayed tumor onset in the *NIC capns1* KO mice.

STAT-1 behaves as a tumor suppressor through promotion of apoptosis as well as inhibition of angiogenesis, tumor growth, and metastasis [54]. A role for STAT-1 in suppression of epithelial to mesenchymal transition has also been described [55]. Increased pY701-STAT-1 observed in *NIC capns1* KO tumors could therefore reflect a role for calpain in regulating these tumor suppressor functions.

EGFR represents an important target since it is often dysregulated in human cancers. EGFR forms heterodimers with HER2 and contributes to survival and proliferative signaling in response to EGF [56]. Enhanced phosphorylation of the EGFR activation loop Y845 residue in *capns1* KO tumors indicated a higher level of activated EGFR (Figure 2). Both EGFR [57] and HER2 [19] have been identified as substrates of calpain, therefore this observation may simply be a reflection of an accumulation of more mature EGFR in the absence of calpain. Given the importance of this signaling node in HER2 tumorigenesis, we focused further study on it using the *capns1 floxed* MTEC model system. There was significantly more of the presumptive mature ~150 kDa EGFR species in *capns1* KO MTEC cells (Figure 7A, 7B), which supports a role for calpain-1/2 in regulating EGFR levels. While this presumptive mature EGFR is rapidly downregulated after EGF treatment in both *capns1* KO and WT MTECs, more of this 150 kDa band persists in KO cells, and more of a faster migrating 135kDa band was observed in the WT cells. It was tempting to predict that this smaller species arose by calpain cleavage of EGFR, since both EGFR [57] and HER2 [19] have been identified as a substrates of calpain. However, it is more likely that this 135 kDa band corresponds to immature EGFR in the ER/Golgi compartment. A prominent band at ~100 kDa was detected with antibodies to both EGFR and pY845-EGFR (Figure 7A). HER2 has been reported to give rise to a 95 kDa species which is associated with elevated PI3K/AKT signaling and trastuzumab resistance [58–60], and calpain is implicated the generation of intracellular fragments of HER2, as well as the activation of AKT [11, 19, 47]. EGFR is also a known calpain substrate, but the occurrence of this ~100 kDa species in the MTEC system was not correlated with *capns1* status, so it cannot be a uniquely calpain-generated fragment of EGFR. HER2 levels were very low in these MTEC cells, and even after forced ectopic over-expression of HER2, we were unable to correlate the presence of unique HER2 fragments with *capns1* status (unpublished observation).

Interestingly, EGF-induced pY845-EGFR levels persisted for longer in *capns1* KO MTECs (Figure 7A), and this correlated with persistence of activated MEK1/2 and ERK1/2 levels (Figure 7A, 7C, 7D). These observations are somewhat surprising given the delayed tumor onset in the *capns1* KO *NIC* transgenic model (Figure 1) and the complete block in tumorigenic potential in the *capns1* KO MTEC engraftment model (Figure 6 and Supplementary Figure 2). However, delayed tumor onset correlating with increased signaling through the ERK1/2 pathway has been observed previously in the neu^NT^ transgenic mice model and MTEC lines derived from them [61]. Similar observations have been described by others as biphasic behavior of ERK signaling, capable of promoting differentiation or quiescence with high ERK activation vs mitogenesis with lower ERK activation [62–64]. We speculate that this may be a reflection of compensatory signaling in the absence of calpain.

We also show that *capns1* KO in MTECs is associated with increased sensitivity to both doxorubicin and lapatinib (Figure 5). *Capns1* KO tumors arising *in vivo* in the *NIC* mice displayed enhanced activation of EGFR (Figure 2), and this was phenocopied *in vitro* in the MTEC model using EGF stimulation (Figure 7). Enhanced activation of EGFR may reflect a survival response to the absence of calpain. Since lapatinib inhibits both HER2 and EGFR, enhanced lapatinib sensitivity of *capns1* KO MTECs might be attributed to an attenuation of compensatory EGFR signaling when calpain is ablated. In the case of doxorubicin, resistance is mediated in part by expression of Pgp, and we have shown that calpain expression correlates with Pgp expression in MEFs [25]. Furthermore, calpain cleavage of topoisomerase IIα has been shown to promote doxorubicin resistance [36]. In summary, survival responses triggered by challenge with doxorubicin and lapatinib may engage calpain-dependent pathways, which bodes well for the potential of calpain inhibition to enhance the efficacy of these therapeutics.

The most compelling observation in this study was the loss of the tumorigenic potential of MTECs upon *capns1* KO. While we cannot rule out the possibility that *capns1 KO* tumors might have emerged if we had waited longer than 150 days, that was not observed in any of the several dozen late arising tumors from engrafted *capns1 KO* MTECs. Angiogenesis is known to be required for the growth of tumors beyond a size of approximately 2 mm^3^. As calpain cleavage of filamin-A has been implicated in the nuclear localization of HIF1α [39], we initially speculated that failure to trigger the angiogenic switch might account for these results. However, overall production as well as nuclear localization of HIF1α were unaffected by calpain status *in vitro* (Supplementary Figure 3). MMP-2 and MMP-9 production *in vitro* were also unaffected (Supplementary Figure 4). This suggests that the block in tumorigenesis at the orthotopic site might relate to a role for calpain in stromal conditioning. For example, loss of calpain may impact the ability of engrafted MTECs to recruit and promote pro-tumorigenic behavior in cancer-associated fibroblasts [65, 66].

*capns1* KO in this MTEC model did not reduce cell migration in a scrape-wound assay (Figure 4A); however, *capns1* KO was associated with attenuated invasive behavior in matrigel-coated Boyden chamber assays (Figure 4B). This is interesting since *capns1* KO in MEFs has previously been associated with defects in cell migration [15] as well as invasion [17]; and *capns1* knockdown in MDA-MB-231 cells correlated with reduced migration and invasion [25]. Calpain has also been linked with lamellipodia dynamics at the leading edge and focal adhesion turnover at the trailing edge of fibroblasts [16, 18]. We were surprised that loss of calpain-1/2 in the MTEC system did not attenuate their migration behavior, yet defective invasive behavior was conserved. MMPs are important players in cell invasion, and we have previously linked calpain to MMP production [17]. EGFR and PI3K-AKT signaling has been implicated in the production of MMPs [67] and although we were unable to identify differential levels of active MMP-2 and MMP-9 by gelatin zymography (Supplementary Figure 4), this assay is limited, as it dissociates tissue inhibitors of metalloproteineases (TIMPs), thus we cannot exclude the possibility that calpain participates elsewhere in the MMP activation cascade [68].

In conclusion, our *in vivo* results demonstrate that loss of calpain-1/2 delays spontaneous tumor onset in a model of HER2^+^ tumorigenesis but does not prevent progression. The possibility that other CAPNS1-independent calpain isoforms could provide redundant functions to potentiate the delayed tumor onset has not been addressed in this study. Phosphoproteomic analysis suggests that tumors arising in the presence of calpain-1/2 engage in subtly different pro-tumorigenic signaling than tumors arising in the absence of calpain-1/2. Differences in the phosphorylation states of EGFR, GSK-3β, JNK, MARCKS and STAT-1 in *capns1* KO tumors represent important leads for future studies aimed at elucidating the molecular involvement of calpain in HER2^+^ breast cancers. Most significantly, calpain was critically required for tumorigenesis in an orthotopic engraftment model, suggesting its inhibition might be an effective treatment strategy. Finally, we show that KO of *capns1* enhances cytotoxicity mediated by doxorubicin and lapatinib. Taken together, these results suggest that inhibition of calpain in established malignancies may confer therapeutic benefits and may also enhance the efficacy of currently employed therapeutics like doxorubicin and lapatinib. This underscores the need to develop clinically effective pharmacological inhibitors of calpain-1/2.

## MATERIALS AND METHODS

### Spontaneous tumor development and reverse-phase protein array analysis

Transgenic mice expressing oncogenic rat HER2/NEU and CRE-recombinase in mammary epithelial cells from a bicistronic transcript under the control of the mouse mammary tumor virus promoter (*MMTV-NEU-IRES-CRE/*otherwise known as *NIC*) [28] were crossed with *capns1 floxed* mice [6] to produce compound *NIC capns1^flox/flox^* or *NIC capns1^+/+^* females. Mammary glands were palpated weekly by an individual who was blinded to mouse genotypes to determine the age when spontaneous tumor formation occurred. At endpoints, mice were euthanized, tumors resected, snap frozen in liquid N_2_ and prepared for reverse-phase protein array (RPPA) analysis using recipe 10 and a panel of 128 antibodies as described [29, 30].

### Cell line derivation

Mammary tumor epithelial cells (MTECs) were derived from *neu^NT^* transgenic mice (strain TG.NK obtained from Jackson Labs) expressing oncogenic rat HER2/NEU in mammary epithelial cells under the control of the mouse mammary tumor virus promoter [31] crossed with *capns1*^flox/flox^ mice [6]. A tumor from a *neu^NT^ capns1^flox/flox^* female mouse was excised and a cell suspension was developed by mechanical disruption and digestion with 0.25% trypsin, followed by culture in improved minimum essential medium (IMEM, Corning) supplemented with 1% fetal bovine serum (FBS; Sigma), 10 μg/mL insulin (Sigma), 15 ng/mL EGF (Sigma), 1 μg/mL hydrocortisone (Sigma), and 20 nM estrogen (Sigma) as described [61, 69] until an immortalized cell line was achieved. The FBS concentration for immortalized cells was increased to 5% and supplemented with insulin, EGF and hydrocortisone. Estrogen was discontinued after 10 passages, and immortalized cells were subsequently cultured in IMEM, supplemented with 5% FBS, and 1% antibiotic-antimycotic (AA; Gibco) (“complete IMEM”). MTECs were maintained in a humidified incubator at 37.5°C, 5% CO_2_.

To produce *capns1* knockout (KO) and vector control (WT) MTECs, 2.5 × 10^5^ cells were seeded on a 60 mm dish (Sarstedt) and transduced with retrovirus expressing CRE recombinase or an empty vector [6] in a pMSCV-puro vector (Clontech). Puromycin (Gibco) was used as a selectable marker, and disruption of the floxed *capns1* gene was validated by CAPNS1 immunoblotting and HEPES-imidazole casein zymography. Cells surviving culture in 2 μg/mL puromycin were lysed into radio-immunoprecipitation assay buffer for immunoblotting (RIPA; 50 mM Tris-HCl, 150 mM NaCl, 1% NP-40, 0.1% SDS and 0.5% sodium deoxycholate supplemented with protease and phosphatase inhibitors 1 mM sodium orthovanadate (Sigma), 1 mM phenylmethane sulfonyl fluoride, 5 μg/mL leupeptin (Sigma), and 2 μg/mL aprotinin (Sigma)); or lysed into HEPES/Triton X-100 lysis buffer for HEPES-imidazole casein zymography as described [70]. Lysates were clarified by centrifugation (12, 000 × g for 15 minutes at 4°C), and protein quantification was performed using the Pierce bicinchoninic acid (BCA) kit (Thermo Scientific). *Capns1* KO was validated by immunoblotting using an in-house rabbit polyclonal antibody generated against bacterially expressed rat calpain-2, which detects murine CAPN2 and CAPNS1 polypeptides [71], and by casein zymography to assess calpain-1 and calpain-2 enzymatic activity. Lysates from *capns1* KO or WT mouse embryonic fibroblasts (MEFs) [6] were used as controls for immunoblotting and zymography.

### Migration, invasion, chemosensitivity, hypoxic response, and EGF signaling analysis

To assess migration, 1.5 × 10^4^ WT or *capns1* KO MTECs were seeded in sextuplicate on 96-well ImageLock plates (Essen Bioscience) and allowed to adhere overnight. The following day cell monolayers were scrape-wounded using the IncuCyte Wound Maker (Essen Bioscience) and washed twice with media. Cells were then cultured in 100 mL of complete IMEM and placed in an IncuCyte system (Essen Bioscience) maintained at 37°C, 5% CO_2_ and imaged with a 10× objective every 2 h until the wounds had closed.

For cell invasion assays, 8 μm-pore transwells (Corning #353097) were coated with 15 μg of growth-factor reduced matrigel (VWR) in 25 μL of serum-free IMEM and allowed to dry overnight. The following day, the matrigel was rehydrated with serum-free IMEM for 2 h in a humidified incubator at 37°C, 5% CO_2_. One × 10^5^ WT or *capns1* KO cells were added in triplicate wells to the top chambers in serum-free IMEM. Media in the lower chambers was replaced with complete IMEM. Cells were allowed to invade for 24 h, then non-invading cells were removed from the top chambers with cotton swabs, and invading cells in the lower chambers were fixed in ice-cold methanol and stained with DAPI (Sigma) and imaged under a 10× objective.

To assess cytotoxicity, 2 × 10^4^ WT or *capns1* KO MTECs were seeded in 96-well plates (Grenier Bio-One) in triplicate and allowed to adhere overnight. Increasing concentrations of doxorubicin (Sigma) or lapatinib (Toronto Research Chemicals) were added to the wells and cells cultured for 72 h. At endpoint, the PrestoBlue reagent (ThermoFisher) was utilized to quantify cell viability by measuring fluorescence on a SpectraMax M2 plate reader (590 nm). Chemosensitivity experiments were analyzed using Prism Graphpad.

Hypoxic response was assessed by seeding 1.5 × 10^6^ WT or *capns1* KO MTECs in 10 cm dishes (Sarstedt) and allowing cells to adhere overnight in complete IMEM. The following morning cells were washed once with PBS and changed to serum-free IMEM. Positive controls were treated with 100 μM of cobalt chloride and half of the dishes were transferred to a hypoxic incubator (0.1% O_2_). After 24 hours cells were placed on ice, rinsed once with PBS and lysed into RIPA (as described previously) or nuclear extraction buffer (20 mM HEPES) pH 7.4, 10 mM KCl, 2 mM MgCl_2_, 1 mM EDTA, 1 mM EGTA, 1 mM DTT supplemented with protease and phosphatase inhibitors as previously described. For nuclear extracts, cells were collected with a cell scraper and passed through a 27-gauge needle 10 times. Lysates were then incubated on ice for 20 minutes and centrifuged 720 × g for 5 minutes at 4°C. The pellet was then washed once in 500 μL of nuclear extraction buffer and passed through a 25-gauge needle 10 times, followed by centrifugation for 10 minutes at 720 × g. The supernatant was discarded, and the pellet was resuspended in 2× SDS sample buffer (100 mM Tris-HCl pH 6.8, 0.2% bromophenol blue, 20% glycerol, 200 mM DTT) and boiled for 5 minutes. Protein quantification was performed using the DC Protein Assay (BioRad) according to the manufacturer's instructions. Immunoblotting was performed, and samples probed for HIF1α (Cell Signaling CST# 36169), α-tubulin (Sigma Cat# T6199), and nuclear lamin B1 (Cell Signaling CST# 12586).

Response to EGF stimulation was assessed by seeding 7.5 × 10^4^ WT or *capns1* KO MTECs in 60 mm dishes overnight in complete IMEM. MTECs were washed once in PBS and changed to serum-free IMEM overnight, then stimulated with 3 mL of 50 ng/mL EGF (Thermo Fisher) prepared in serum-free IMEM for the indicated times. Cells were placed on ice and washed once with PBS containing 1 mM sodium orthovanadate and lysed into RIPA as previously described. Immunoblotting was performed, and samples probed for EGFR (Cell Signaling CST# 2232), P-EGFR (Y845) (Cell Signaling CST# 2231), MEK (Cell Signaling CST# 9122), P-MEK (S217/221) (Cell Signaling CST# 9121), ERK (Cell Signaling CST# 9102), and P-ERK (T202/Y204) (Cell Signaling CST# 9101).

### Matrix metalloproteinase assay

To assess the secretion of matrix metalloproteinase 2 and 9 (MMP2/9), 7 × 10^5^ WT or *capns1* KO MTECs were seeded overnight on 60 mm tissue culture dishes in complete IMEM, and the following day plates were washed twice with PBS and overlaid with 3 mL of serum-free IMEM. Cells were allowed to condition the medium for 48 h and gelatin zymography was performed as described by [72].

### Orthotopic tumor growth

One × 10^6^ WT or *capns1* KO MTECs were suspended in 50 mL of 50% phosphate-buffered saline, and 50% growth factor reduced matrigel (VWR), and engrafted into the number four (abdominal) mammary gland of *BalbC-Rag2*^−*/*−^*/IL2R*γ*c*^−*/*−^ mice. After 7 d, staples were removed from surgical sites and tumor volumes assessed until endpoints. At endpoints, mice were euthanized, and tumors resected and lysed into RIPA buffer containing protease and phosphatase inhibitors (as described previously) for calpain immunoblotting analysis. Mice were housed at the Queen's University Animal Care Facility, and all procedures were carried out in accordance with the guidelines specified by the Canadian Council on Animal Care, with approval from the University Animal Care Committee.

### Reverse-phase protein microarray (RPPA)

Cell lysates prepared from various tumor samples were printed using Aushon 2470 Arrayer (Aushon Biosystems). Validation of antibodies, staining, and analysis of array data was performed as described previously [29, 30].

## SUPPLEMENTARY MATERIALS FIGURES



## Abbreviations

AKT: protein kinase B
CRE: causes recombination
EGF: epidermal growth factor
EGFR: EGF receptor
ERK: extracellular signal-regulated kinase
floxed: flanked by loxP recombination sites
GSK3β: glycogen synthase kinase 3 beta
HER2: human epidermal growth factor receptor 2
HIF1α: hypoxia-inducible factor 1 alpha
IRES: internal ribosome entry site
JNK: Jun N-terminal kinase
KO: knockout
MAPK: mitogen activated protein kinase
MARCKS: myristoylated alanine-rich C-kinase substrate
MEF: mouse embryonic fibroblasts
MEK: MAPK kinase
MMP: matrix metalloprotease
*MMTV-LTR*: mouse mammary tumor virus long terminal repeat
MRP2: multidrug resistance protein 2
MTEC: mammary tumor epithelial cell
MYC: myelocytomatosis viral oncogene
NEU: neuroblastoma oncogene
NIC: *MMTV-NEU-IRES-CRE* transgene
Pgp: P-glycoprotein
PI3K: phosphatidylinositol-3-kinase
PKC: protein kinase C
PP2A: protein phosphatase 2A
STAT1: signal transducer and activator of transcription 1
