# Supplementary material for: Genetic disruption of calpain-1 and calpain-2 attenuates tumorigenesis in mouse models of HER2+ breast cancer and sensitizes cancer cells to doxorubicin and lapatinib
Source: Oncotarget. 2018 Sep 7;9(70):33382–95. doi: 10.18632/oncotarget.26078 (PMC6161787; doi:10.18632/oncotarget.26078)
Supplement: Supplementary file 1 [file oncotarget-09-33382-s001.pdf]

Genetic disruption of calpain-1 and calpain-2 attenuates tumorigenesis in mouse models of HER2+ breast cancer and sensitizes cancer cells to doxorubicin and lapatinib

SUPPLEMENTARY MATERIALS

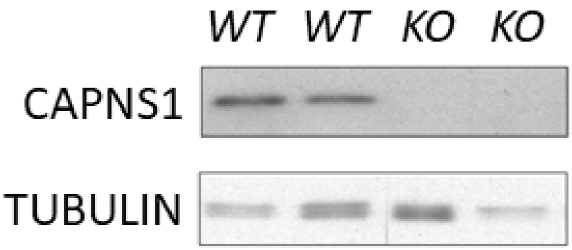

Supplementary Figure 1: CAPNS1 expression is ablated in mammary tumors from NIC *capns1$^{flox/flox}$* mice. Tumor lysates from NIC *capns1$^{flox/flox}$* (*KO*) or NIC *capns1$^{+/+}$* (*WT*) female mice were assessed for CAPNS1 or tubulin expression by immunoblotting.

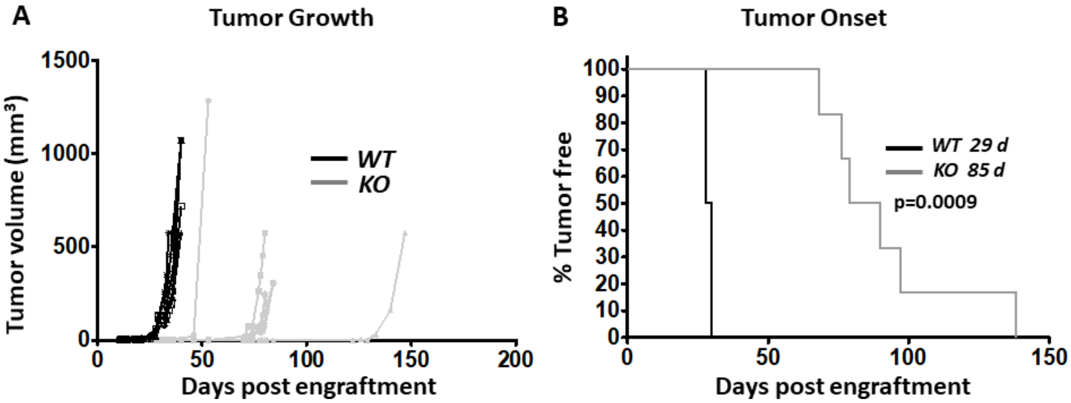

Supplementary Figure 2: *capns1 KO* is associated with reduced tumorigenic potential. One × 10^6 capns1 *KO* or *WT* MTECs were engrafted into the number four mammary glands of female *Rag2$^{-/-}$* I*L-2Rγc$^{-/-}$* mice ($n = 6$ for each cohort). (**A**) Caliper measurements of tumor volumes. (**B**) Median time to onset of palpable tumors at the engraftment site were 85 or 29 days for *KO* and *WT*, respectively ($p = 0.0009$, Log-rank (Mantel-Cox) test). All tumors arising in the KO group displayed expression of *capns1* (data not shown).

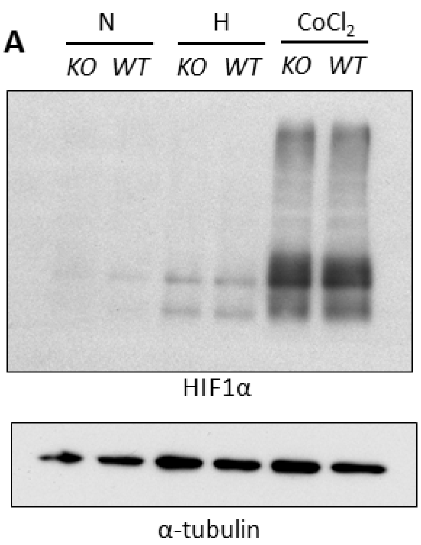

Supplementary Figure 3: *capns1* knockout does not affect hypoxia-induced stabilization of HIF1α. One and a half × 10^6 *capns1* KO or *WT* MTECs were seeded for 24 h under conditions of hypoxia (H) (0.1% O_2), normoxia (N) (21% O_2) or challenge with $CoCl_2$ (100 µM). Lysates were collected and levels of HIF1α were assessed by immunoblotting. Tubulin and nuclear Lamin B1 immunoblotting was performed to assess sample loading. (**A**) Whole cell lysate immunoblotting for HIF1α (**B**) Nuclear enriched immunoblotting for HIF1α.

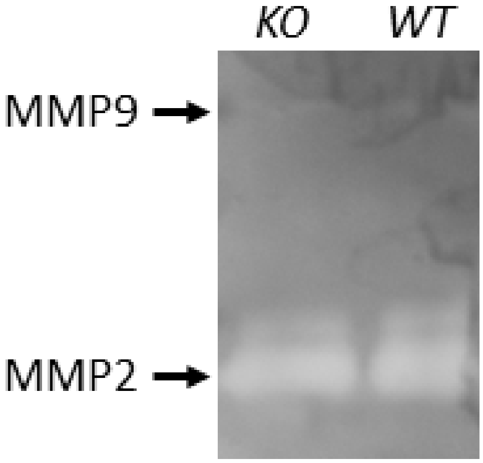

Supplementary Figure 4: *capns1 KO* does not affect secretion or activation of matrix metalloproteinases 2 and 9. Seven × 10^5 *capns1 KO* or *WT* MTECs were seeded and cultured overnight, then changed to serum-free medium the following day. Conditioned media was collected after 24 hours and gelatin zymography performed to assess MMP activity.
